# Expected for acquisition movement exercise is more effective for functional recovery than simple exercise in a rat model of hemiplegia

**DOI:** 10.1186/2193-1801-2-517

**Published:** 2013-10-07

**Authors:** Satoshi Ikeda, Akihiko Ohwatashi, Katsuhiro Harada, Yurie Kamikawa, Akira Yoshida

**Affiliations:** Department of Rehabilitation and Physical Medicine, Graduate School of Medical and Dental Sciences, Kagoshima University, 8-35-1 Sakuragaoka, Kagoshima-shi, Kagoshima, 890-8544 Japan; School of Medical Sciences, Faculty of Medicine, Kagoshima University, 8-35-1 Sakuragaoka, Kagoshima-shi, Kagoshima, 890-8544 Japan

**Keywords:** Stroke, Animal model, Rehabilitation, Functional recovery

## Abstract

**Background and purpose:**

The use of novel rehabilitative approaches for effecting functional recovery following stroke is controversial. Effects of different but effective rehabilitative interventions in the hemiplegic patient are not clear. We studied the effects of different rehabilitative approaches on functional recovery in the rat photochecmical cerebral infarction model.

**Methods:**

Twenty-four male Wistar rats aged 8 weeks were used. The cranial bone was exposed under deep anesthesia. Rose bengal (20 mg/kg) was injected intravenously, and the sensorimotor area of the cerebral cortex was irradiated transcranially for 20 min with a light beam of 533-nm wavelength. Animals were divided into 3 groups. In the simple-exercise group, treadmill exercise was performed for 20 min every day. In the expected for acquisition movement-training group, beam-walking exercise was done for 20 min daily. The control group was left to recover without additional intervention. Hindlimb function was evaluated with the beam-walking test.

**Results:**

Following cerebral infarction, dysfunction of the contralateral extremities was observed. Functional recovery was observed earlier in the expected for acquisition training group than in the other groups. Although rats in the treadmill group recovered more quickly than controls, the beam-walking group had the shortest overall recovery time.

**Conclusions:**

Exercise facilitated functional recovery in the rat hemiplegic model, and expected for acquisition exercise was more effective than simple exercise. These findings are considered to have important implications for the future development of clinical rehabilitation programs.

## Background

Recently, the importance of the therapeutic approach to functional recovery following stroke has received some attention (Zorowitz and Brainin 2011). Constraint-induced movement improved the upper extremity function in patients with mild-to-moderate stroke (Reiss et al. 2012). Treadmill training with body weight support resulted independent ambulation in more patient than control however the results were not statistically significant (Ada et al. 2010). One recent meta-analysis indicated that increased duration or intensity of therapeutic exercise was associated with improvement in the recovery of function in clinical patients (Kwakkel et al. 2004). Here, we report the effects of rehabilitative approaches on functional outcomes after photochemically induced stroke. In a rat model of ischemia, exercise improved functional outcomes while involuntary exercise and forced exercise did not facilitate motor recovery, as they did in the controls (Ke et al. 2011). In another rat stroke model, a significant difference was found at 1 week post-infarction between the non-exercise group and the exercise group on foot fault test scores (Yang et al. 2012). In clinical practice, patients received various types of rehabilitative therapy depending on the various levels of experience and ability of therapists. However, these studies effective rehabilitative approaches compare to non rehabilitation group or not effective approaches within same interventive duration.

It is unclear that the effect of different two effective rehabilitation strategies on functional recovery, matched for duration, produces different outcomes. We studied the effect of different rehabilitative approaches to functional recovery by using a rat photochemical cerebral infarction model.

## Methods

The study was carried out in accordance with the Guide for Animal Experimentation of faculty of Medicine of Kagoshima University and the US National Institute of Health guidelines. These animals were housed in plastic cages in the room controlled environmental conditions with a 12–12 hour light–dark cycle and had free access water and standard foods.

Twenty-four male Wistar rats aged 8 weeks were used. The cranial bone was exposed under deep anesthesia (sodium pentobarbital, 40 mg/kg). Photochemical infarction was induced using the method originally established by Watson et al. (Watson et al. 1985). This method has been widely adopted because it does not require a craniotomy. Rose bengal is a photosensitive dye, which activates platelets under irradiation by a green beam of light, resulting in localized thrombosis. Rose bengal was injected intravenously (20 mg/kg), and the sensorimotor area of the cerebral cortex was irradiated transcranially for 20 min. Irradiation was performed using green light (533-nm wavelength) and a fiber optic light guide of 10 mm diameter (MHF-G150LR, MSG10-1100S; Moritex, Tokyo) (Horinouchi et al. 2007 Ohwatashi et al. 2013). Animals were subsequently divided into 3 groups. In the simple-walking exercise group, animals walked on a treadmill at a rate of 8 m/min for 20 min per day. As expected for acquisition movement exercise group, animals were walked across an elevated narrow beam (122 cm long × 2.5 cm wide) for 20 min every day. Control animals were allowed to move about freely. The locomotion of infarcted rats was evaluated using the same beam-walking task mentioned above. Locomotor performance was quantified as a functional recovery score. Feeney’s assay is used for many studies. Former studies indicated the functional ceiling is obtained untill 14 days from infarction and normal control showed score 7 (Feeney et al. 1982 Abo et al. 2001). Two weeks after infarction, animals were perfused transcardially with 300 mL of 4% paraformaldehyde in 0.1 M phosphate buffer at pH 7.4 under deep anesthesia. The brains were removed and postfixed in the perfusion solution overnight, and the infarct surface area (mean ± standard deviation) was measured. Statistical analysis was performed using one-way analysis of variance, followed by post-hoc tests (Fisher’s PLSD) to compare the three groups.

## Results

**Figure 1:**
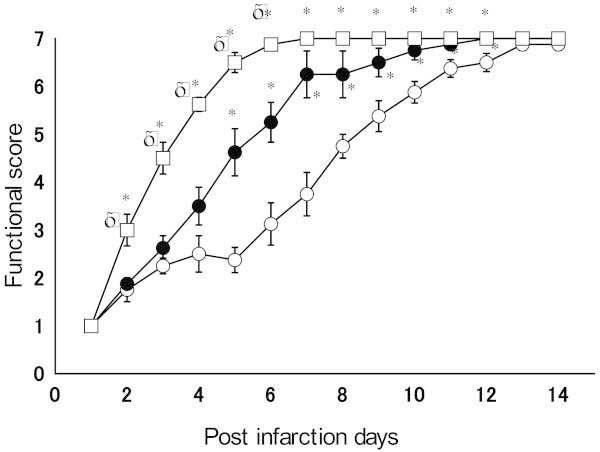
**Functional score (mean ± SE) after photochemical infarction in the expected movement exercise group (squares), treadmill-exercise group (close circles), and non-exercise control group (open circles) are shown.** Significant differences (*P* < 0.05) were observed between the control group and each exercise group (*), and between the expected movement exercise group and the treadmill group (†).

Following photochemical ischemia, dysfunction of the contralateral extremities was observed. Functional recovery was observed earlier in the expected for acquisition movement exercise group than in the other two groups. Rats in the treadmill group also recovered earlier than the controls did. Figure 1 shows functional recovery of the photochemically infarcted rats. Open circles represent the control group, closed circles represent the simple-walking exercise group, and squares represent the expected for acquisition movement exercise group. Animals achieved a score of 1 on the day following infarction, and functional recovery progressed day by day. Recovery was remarkable at 2–4 days after infarction, and the expected for acquisition movement exercise group achieved a score of 7 on day 7. The score was significantly higher than that of the simple-walking exercise group from the second day to the sixth day following infarction, and higher than that of the controls from the second day to the twelfth day.

**Figure 2:**
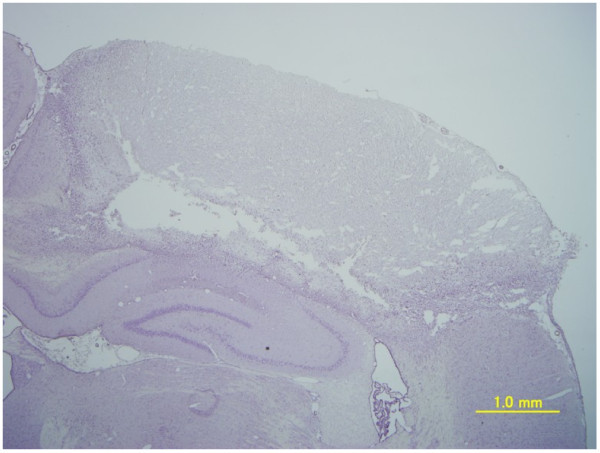
**Photomicrograph of the section of the rat infarcted cortex with Hematoxylin staining.**

The simple-walking exercise group showed evidence of recovery significantly earlier than the control group did, with higher recovery scores from the sixth day to the twelfth day. Locomotor performance was remarkable 4–7 days after infarction, and the group score reached 7 by day 11. In the control group, recovery accelerated 6–10 days after infarction, and animals achieved a score of 7 by approximately 2 weeks after the infarction. There are infarcted lesion in the irradiated cerebral cortex (Figure 2). The infarct area measured 35.1 ± 2.12 mm^2^ in the control group, 42.1 ± 2.33 mm^2^ in the simple-walking exercise group, and 37.1 ± 3.16 mm^2^ in the expected movement exercise group. There were no significant differences between infarct areas among the experimental groups.

## Discussion

There have been many investigations of rehabilitation after stroke (Kwakkel et al. 2004 Ke et al. 2011 Abo et al. 2001 DeBow et al. 2003 Marin et al. 2003). However, the relative benefits of contrasting approaches have not been firmly established (DeBow et al. 2003). Ke et al. reported that voluntary exercise improves motor function, while involuntary and forced exercise does not improve the motor function (Ke et al. 2011).

The constraint-Induced movement therapy and rehabilitation exercise lessen motor deficits in the rat hemorrhagic stroke model (DeBow et al. 2003). Voluntary exercise improved neurobehavioral outcomes after ischemic brain injury in the rat (Marin et al. 2003). In the clinical patient, high-intensity rehabilitation therapy appeared to facilitate recovery (Kwakkel et al. 2004). However, these reports did not indicate different effective rehabilitative approaches.

Our results indicate that both simple-walking exercise and beam walking accelerate functional recovery compared to natural recovery. As expected for acquisition movement exercise was more effective than simple-walking exercise for improving beam-walking performance. The difference in effectiveness between beam-walking and treadmill exercisemay derive from the difference in task difficulty, rather than task similarity. There is a possibility that beam –walking task needs delicate control of hindlimb rather than treadmill walking. Therefore treadmill training is thought to affect the functional recovery of beam walking task less than beam walking training. It is difficult to strictly discriminate motor recovery from motor learning. However, in clinical rehabilitation, functional training is thought that the sum of spontenous neuronal recovery and motor relarning and neuronal reorganization by exercise (Hosp and Luft 2011). Our result suggested that beam walking exercise has more accelerative effect than treadmill exercise in functional recovery.

These findings suggest that rehabilitative effects depend on the specific rehabilitative approach; exercises that reproduce the demands of a specific task should be more effective than simple exercise at improving performance on that task. Consequently, therapy that anticipates a patient’s specific functional requirements following cerebral infarction would be more effective than simple exercise for achieving functional recovery.

## Conclusions

Rehabilitative exercise facilitated functional recovery in our rat hemiplegic model, and different exercises had different effects on functional recovery. A rehabilitative approach that expected functional requirements led to a better functional recovery. These findings are considered important for developing clinical rehabilitation strategies for functional recovery.
